# Estimating Risks of Heat Strain by Age and Sex: A Population-Level Simulation Model

**DOI:** 10.3390/ijerph120505241

**Published:** 2015-05-18

**Authors:** Kathryn Glass, Peter W. Tait, Elizabeth G. Hanna, Keith Dear

**Affiliations:** 1 National Centre for Epidemiology and Population Health, Australian National University, Canberra 2601, Australian; E-Mails: aspetert@bigpond.com (P.T.); Liz.Hanna@anu.edu.au (E.H.); 2 Duke Global Health Institute, Duke Kunshan University, Kunshan 215316, China; E-Mail: keithdear4@gmail.com

**Keywords:** heat storage, simulation model, population-level, MANMO, heat strain

## Abstract

Individuals living in hot climates face health risks from hyperthermia due to excessive heat. Heat strain is influenced by weather exposure and by individual characteristics such as age, sex, body size, and occupation. To explore the population-level drivers of heat strain, we developed a simulation model that scales up individual risks of heat storage (estimated using Myrup and Morgan’s man model “MANMO”) to a large population. Using Australian weather data, we identify high-risk weather conditions together with individual characteristics that increase the risk of heat stress under these conditions. The model identifies elevated risks in children and the elderly, with females aged 75 and older those most likely to experience heat strain. Risk of heat strain in males does not increase as rapidly with age, but is greatest on hot days with high solar radiation. Although cloudy days are less dangerous for the wider population, older women still have an elevated risk of heat strain on hot cloudy days or when indoors during high temperatures. Simulation models provide a valuable method for exploring population level risks of heat strain, and a tool for evaluating public health and other government policy interventions.

## 1. Introduction

Climate projections suggest that mean global temperature will likely increase by between 1.1 and 4.8 degrees Celsius by 2081–2100 when compared to 1986−2005 [1], with multiple implications for human health [2,3]. One of the direct globally observed effects of warming is an increased risk of mortality and morbidity due to thermal stress [4,5,6]. Average global warming poses a lower heat-related health risk than the associated increase in extreme heat events, as seen in the 70,000 deaths in Europe in 2003, and 55,000 in 2010. Humans maintain their core temperature within a very narrow range through a suite of thermoregulatory mechanisms. Heat strain arises when thermoregulatory capacity is overwhelmed, in situations where heat exposure and heat generation exceed heat loss. While adaptation and preventative measures can reduce the impact of extreme temperatures [5,6], the severity and duration of heat events occurring globally may exceed the capacity for humans to inhabit many currently populated regions [7]. Further, widespread reliance on protective measures such as air conditioning may leave vulnerable populations at risk due to limitations of accessibility [8,9].

Understanding individual risks due to thermal stress is complicated by specific combinations of age- and gender-related differences in heat response [10,11] and differences in heat exposure patterns. Unsafe heat exposure can occur in the home [12], during recreation [13,14], and at work [15], with work-related exposures raising concerns for both health and productivity [16]. Population health objectives include harm minimization via identification and initiation of protective measures that can be implemented at either the individual or the population level. These are likely to have different uptakes in different groups [17]. In order to fully understand the impacts of these prevention measures, we must scale up from individual behavior to entire populations.

Mechanistic simulation models provide a new and valuable tool for understanding population-level impacts of heat strain. Such models are applied widely in ecology and infectious disease epidemiology [18] to model individual behavior, and to analyze the impact of multiple concurrent interventions [19,20]. They have also proven valuable in social science research to help formalize complex theories [21] and have been usefully applied to various business problems ranging from modeling individuals escaping fires to comparing business models for internet service providers [22]. For many decades, mathematical models of the human thermal response have been used to simulate human physiological and thermoregulatory responses under different environment conditions and activity levels [23,24,25]. These show good correlation with empirical studies [26,27], however, many were predominantly developed for normal thermal conditions [23], or developed from sample sizes as low as four individuals [28,29]. Problematic for models is the variation of thermoregulatory responses between different individuals, even under the same environmental conditions. In recent years, information on age, gender, and body-mass-based differentials in heat response has emerged [30,31,32].

Most research on human thermoregulation has focused on fit young adult males [33], although the research focus has expanded over recent years to include the young [34], the old [35], and females [36]. A key gap in the literature is a population-level model of health risk from heat storage in the body leading to heat stress under a warming climate. Here, we imbed a widely-used individual-level model—The MANMO model of human heat balance [24,37,38]—Into a mechanistic simulation model of the Australian population to explore the influence of weather and individual characteristics on population-level risks of heat strain.

## 2. Materials and Methods

**Figure 1:**
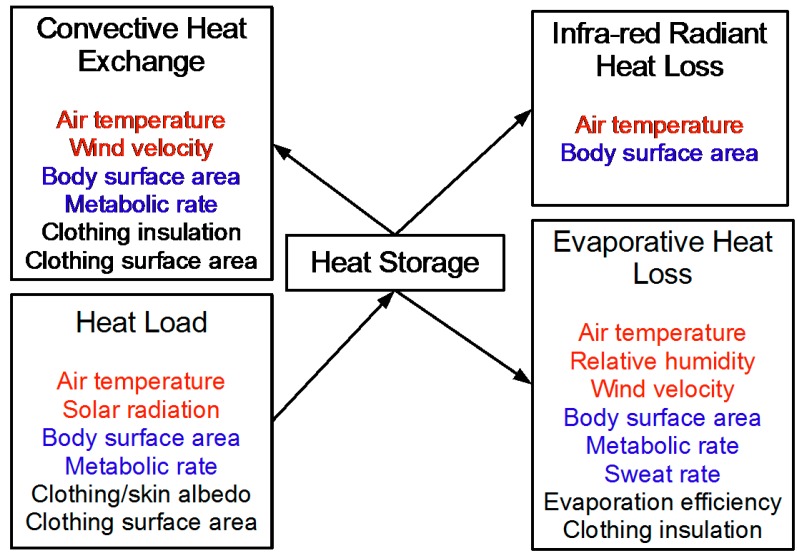
The MANMO model showing heat flow (loss or gain), with weather inputs in red, individual characteristics in blue, and fixed values in black. Weather conditions are sourced from the Australian Bureau of Meteorology. Body surface area is a function of height and weight, which in turn is driven by age and sex. Metabolic rate varies according to age and sex characteristics, as outlined in the Supplementary Material (Figure S1 and Table S1). Sweat rates are assumed to vary by sex and age as described in the text. Fixed values are listed in Table 1.

At the individual level, heat storage was estimated using Myrup and Morgan’s MANMO (“man model”) [24], as implemented and applied by Maloney and Forbes [39]. The input variables for the MANMO model consist of weather variables (air temperature, solar radiation, relative humidity, and wind velocity) together with individual level characteristics (body surface area, metabolic rate, sweat rate) that depend on age and sex. Figure 1 provides a diagram of this model, with individual characteristics in blue, and weather variables in red. For simplicity, some model parameters were fixed at values listed in Table 1. We further adjusted the Maloney and Forbes implementation of the MANMO model for clothing temperature to better reflect both indoor and outdoor exposures.

**Table 1:** Fixed values in the MANMO model [24] as implemented by Maloney and Forbes [39]. All are dimensionless unless otherwise indicated in the table. Figure 1 indicates the role that these values play in the model.

Characteristic	Description	Value
Clothing albedo *	reflecting power of clothing	0.3
Clothed proportion	proportion of body covered	0.4
Clothing surface area ratio	factor increase in surface area for clothed area	1.08
Evaporation efficiency	proportion of sweat that evaporates	0.85
Insulation of clothing **	basic dry thermal insulation of clothing	0.093 m^2^∙W^−1^∙K
Skin albedo ***	reflecting power of skin	0.3
Skin temperature	kept fixed to simplify sweating model (see text and Maloney and Forbes [39])	36 °C

* Typical values in simulations range from 0.3 to 0.4 [39,40]. ** Using 0.6 clo = 0.6 × 0.155 = 0.093 m^2^∙W^−1^∙K as described in [26]. *** Typically 0.4–0.45 for lighter skin, and 0.2–0.25 for darker skin [40].

### 2.1. Weather Variables

Weather data were sourced from the Australian Bureau of Meteorology published measurements of temperature and humidity at 3:00 p.m., together with daily solar radiation estimates [41]. Although wind speed measurements are available, these are taken above ground level, and so are rarely typical of wind experienced by individuals. We assumed that all individuals experience a wind velocity of 2.5 m∙s^−1^ outdoors (corresponding to a light breeze) and 0.3 ms^−1^ indoors, which corresponds to body movement [42].

### 2.2. Individual Level Characteristics

The three individual-level characteristics that are required for the MANMO model are: body surface area, sweat rate, and metabolic rate. Body surface area in square meters is derived from height in centimeters and mass in kilograms as:

*Surface Area* = 0.007184 *mass*^0.425^*height*^0.725^ (1)

As in Maloney and Forbes [39], we assumed a fairly high value for skin temperature (36 °C) and used maximal sweat rates. These assumptions allowed us to avoid explicitly solving for an equilibrium skin temperature, and eliminate scenarios where evaporative heat loss is overestimated [39]. Maximum sweat rates vary by sex, age, and acclimatization, and have been measured in a number of studies by inducing hyperthermia via either passive exposure or vigorous exercise in participants [43,44,45,46,47,48].

We translated findings from these six studies into the same units as used by the MANMO model (g∙min^−1^∙m^−2^), and fitted a simple linear regression to these data to estimate the contribution of age and sex to maximal sweat rates as:

*Maximum sweat rate* = 7.39 + 2.61 *male* + 1.16 *acclimatization* − 0.07 *age* (2)

That is, we expect maximum sweat rates to decline with age (in years), and to be greater (on average) for males (coded 1) than for females (coded 0). For simplicity, we assumed all individuals are acclimatized or adapted to current temperatures (*acclimatization* coded 1); however, there is scope in latter applications of our model to explore this factor in more detail. Data from pre-pubescent boys indicates that their sweat rates are more in line with those of girls than of older males [49]. We adjusted the model to assume that sweat rates of males and females under 14 years are equal.

We include four levels of exertion: rest, minimal exertion, moderate exertion, and heavy exertion that are linked to occupations types as in Table S1. Metabolic rates are projected from these levels of exertion assuming that metabolic rate increases with body mass (as in [50]), and that a 70 kg individual has metabolic rate of 125 W at rest, 225 W during minimal exertion, 600 W during moderate exertion, and 1280 W during heavy exertion. Note that this assumption influences our analyses of the effect of mass on heat storage in the Supplementary Material.

In addition to the three variables listed above, we also introduced a “weather exposure” variable that indicates individual exposure and the individual’s ability to modify current weather—For example, through the use of air conditioning or shade. We include three categories for this variable: not exposed, indoors exposed, and outdoors exposed. Individuals that are categorized as “not exposed” can modify their weather exposure sufficiently that they do not experience heat stress. The indoor and outdoor categories allowed us to categorize individuals according to exposure to solar radiation, and to assign a wind velocity as described above. This is clearly a broad-brush approach and we acknowledge that there is potential for misclassification of individuals when using employment status to assess exposure. In particular, we do not take account of recreational exposure. See Supplementary Table S1 for a listing of categories relative to occupations.

### 2.3. Scaling from Individuals to Populations

The combination of weather variables and individual level characteristics described above allowed us to measure heat storage in a particular individual on a given day. The aim of our work is to scale up from individuals to model heat storage characteristics in a population on that day. Rather than attempt to simulate heat storage of every individual in the population, we developed a suite of person-types that display the variety of heat risk characteristics in the full population. Each person type includes five individual-level characteristics, namely: weather exposure, mass, height, metabolic rate, and sweat rate. For computational efficiency, continuous variables, such as mass, height and sweat rate are categorized into blocks of 20 kg, 10 cm, and 2 g per square meter per minute, respectively. This is another area for potential later refinement by increasing the granularity of measurement.

To ensure that combinations of mass and height were consistent with the Australian population, we requested a customized report from the Australian Bureau of Statistics giving the proportion of the population in each height-mass pair, by age and sex. Measured heights and weights were obtained from the 2011/12 Australian Health Survey, which includes over 33,000 Australians. In total, this gave us 37 body types across all ages and both sexes. We also requested occupation data across 36 different occupation categories by age and sex, to let us model levels of exertion and weather exposure by age and sex. Details of these categories and our assumptions are provided in the Supplementary Material. Finally, maximal sweat rates were estimated for age and sex categories as described above.

### 2.4. Outcome Variables

The MANMO model provides scope to output a number of possible outcome measures. We chose to follow Maloney and Forbes in assuming a heat capacity of the body of 3.5 J·g^−1^·K^−1^ (as in[39], and then identified the proportion of each age group and sex that gain 2 °C or more in one hour as our main outcome measure [39]. We considered individuals gaining 2 °C or more in one hour under weather conditions at 3:00 p.m. to be at risk of heat stress. Although the proportion of individuals at risk depends on this 2 °C threshold, sensitivity analysis showed that groups at risk of heat strain remained similar with other plausible threshold values.

### 2.5. Model Testing

Although the MANMO model has been used to explore heat storage in individuals [39], our work involves applying the model to a wide range of individual body types and weathers. To ensure that model outputs remain feasible across the range of variables we are considering, we conducted a number of sensitivity analyses of the model with different inputs to confirm that outputs remained plausible. Results of these analyses are reported below and in the Supporting Material.

## 3. Results

Our first step was to explore model behavior at the individual level, varying each input across its plausible range (see Supplementary Figures S2 and S3). We found a slight increase in hourly temperature gain with increasing mass (where height was fixed), and a slight decrease in hourly temperature gain with increasing height (where mass was kept fixed), with findings influenced by our assumptions concerning the impact of mass on exertion levels. Hourly temperature gains increased with exertion levels, and decreased with maximal sweat rates, as expected. We identified different temperature and humidity thresholds for individuals indoors and outdoors, suggesting that heat gain depends on both factors. The MANMO model of clothing is not detailed, and we found relatively little difference in outputs with modification in the proportion of individuals that are clothed.

**Figure 2:**
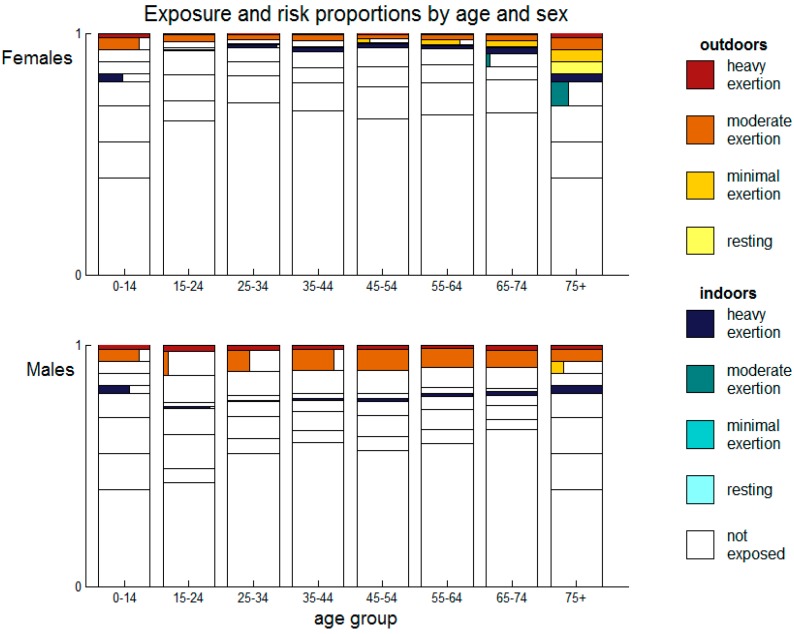
Exposure and risk proportions for each age group and sex at a temperature of 24.9 °C and 62% relative humidity. For each age-sex pair, we show a bar with horizontal lines dividing the bar into the nine rectangles that classify exposure and activity levels as in the legend. Within each rectangle, the proportion of the exposure group that is at risk is shown horizontally. Where the entire rectangle is filled (e.g., males aged 65–74 outdoors moderate exertion) this indicates that the entire exposure category is at risk for that age and sex combination. Where the rectangle is partly filled (e.g., males aged 35–44 outdoors moderate exertion) this indicates that only some of that exposure category is at risk, and that body shape (height and weight) also influences risk in this group under these weather conditions.

Figure 2 summarizes output from the model at a temperature of 24.9 °C with 62% relative humidity—Values for Sydney, Australia on the 1 January, 2014, at 3:00 p.m. The figure shows exposure and activity levels for each sex and age group, together with the proportion of that subcategory that is at risk of heat strain. Each age-sex pair is represented by a bar with horizontal lines dividing it into nine categories of exposure and metabolic rate. Within each rectangle in the bar, the proportion of that exposure group at risk is shown horizontally. An entirely filled rectangle indicates that the entire exposure category is at risk for that age and sex combination, while a partly filled rectangle indicates that only some of that exposure category is at risk, and that risk of heat strain is also influenced by body type—That is by height and weight. Although this figure does not allow cumulative risk levels to be easily gauged, it shows that height and weight are typically less influential in determining heat strain than age, sex, and activity levels, so that many rectangles are either empty or fully filled. In later figures we dispense with this level of detail and present cumulative risk by age and sex.

**Figure 3:**
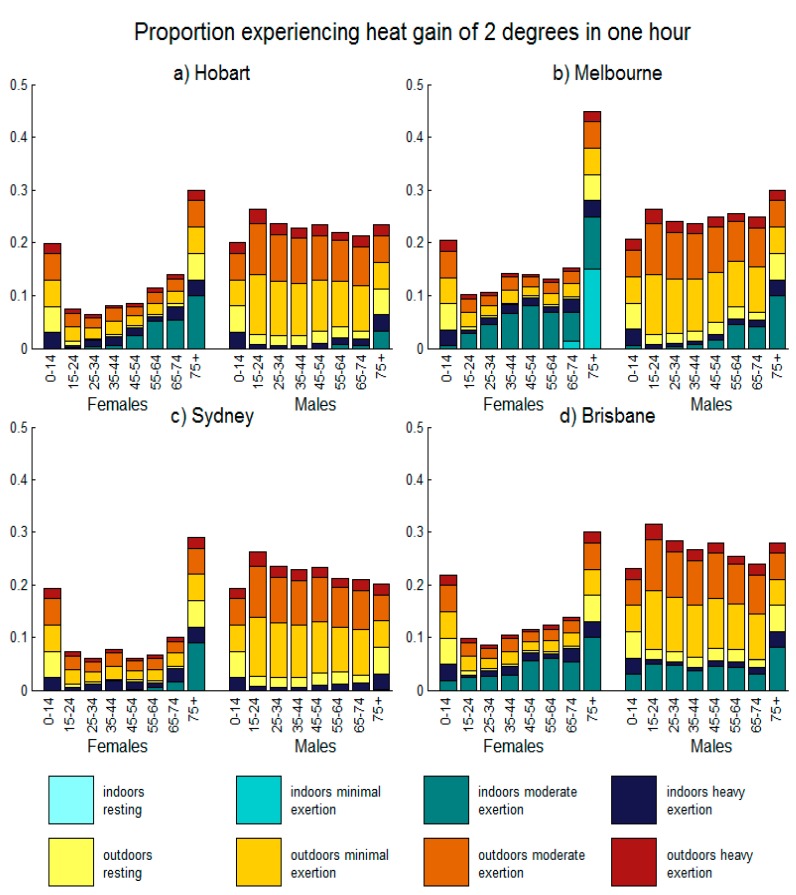
The proportion of each age-sex group that gains 2 °C or more in one hour at weather conditions recorded at 3:00 p.m. on the day with most extreme conditions in January 2014 for each of four Australian cities, assuming no modification in behavior due to the weather. Age and sex groups are classified as those indoors or outdoors, with increasing levels of exertion. For Hobart, the day plotted was the 28 January (36.5 °C temperature, 15% humidity, 595 W∙m^−2^ solar radiation), for Melbourne it was the 16 January (43.2 °C temperature, 16% humidity, 600 W∙m^−2^ solar radiation), for Sydney it was the 29 January (29 °C temperature, 45% humidity, 713 W∙m^−2^ solar radiation), and for Brisbane it was the 4 January (35.5 °C temperature, 47% humidity, 706 W∙m^−2^ solar radiation).

To explore population risks in different climates, we identified days affording high levels of heat stress in January 2014 in each of Australia’s six state capital cities. For comparison, we selected Hobart (latitude 42.9° S), Melbourne (latitude 37.8° S), Sydney (latitude 33.9° S), and Brisbane (latitude 27.5° S) as representing a range of climatic conditions, with humidity increasing closer to the equator. Figure 3 shows the proportion of each age-sex group that gains more than 2°C in one hour at 3:00 p.m. on the day with most extreme conditions in January 2014, assuming no modifications to behavior in response to the weather. Weather conditions on each day are provided in the caption. This figure adopts the same color categories as Figure 2, showing the contribution of different exposure categories to the total proportion of each age and sex category that is at risk of heat strain. Exposure categories are divided into those indoors or outdoors, and by levels of exertion. Unlike Figure 2, we do not include empty bars for individuals not at risk. In most cities, the group at greatest risk is females aged 75 and over, with a very strong effect of age on risk in females.

Risk in males is exacerbated by solar radiation, with men indoors typically only at risk during extreme heat (Melbourne) or when humidity is high (Brisbane). In contrast, there is a clear risk to older females indoors at both moderate and high exertion levels, with risk extending to minimal exertion levels for females aged 75 and older under extreme heat (Melbourne). Children typically have a high surface area to mass ratio, which makes for efficient heat loss when air temperatures are below skin temperatures, however this gradient can be reversed during extreme heat [51]. Likewise, a high surface area to mass ratio can lead to increased heat gain through solar radiation. Figure 3 shows a risk to children on hot, sunny days.

**Figure 4:**
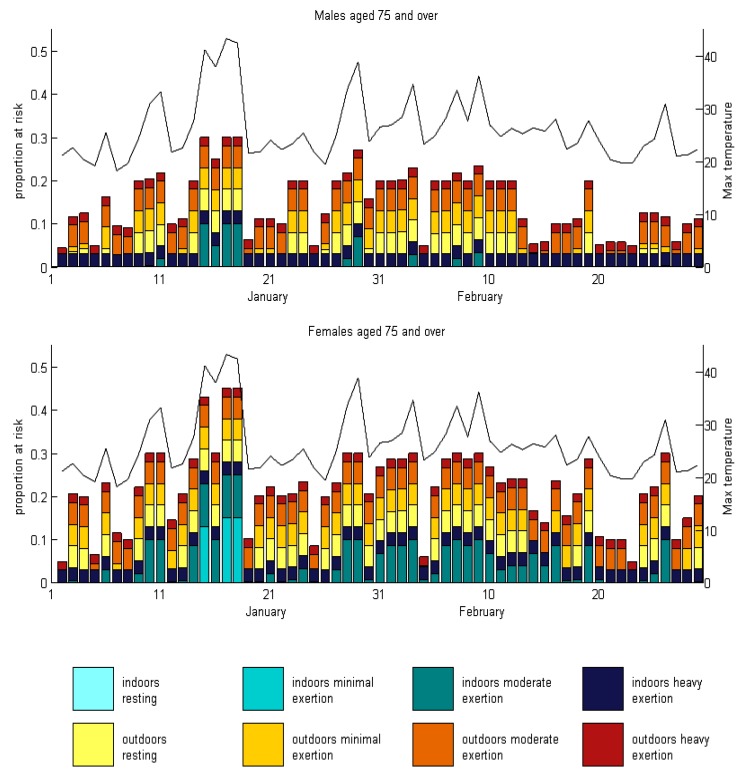
The proportion of individuals aged 75 and over that gain more than 2 °C in one hour at 3:00 p.m. on each day in January and February 2014 in Melbourne, Australia, assuming no modification to behavior. Individuals are categorized by indoor or outdoor exposure, and by their level of exertion. Daily maximum temperature is also shown on each graph (black lines, right hand axis).

In Figure 4, we present the risk of heat strain for males and females aged 75 and over for two summer months (January and February 2014) in Melbourne, Australia, using the same color scheme as in Figure 3. We can see that risks to older females are uniformly higher than risks to older males. Males indoors are only at risk at moderate levels of exertion when temperatures are extreme. In contrast, females indoors at moderate levels of exertion are at risk on most days over this two month period, with even minimal exertion posing a risk during two days where temperatures were over 42 °C.

## 4. Discussion

We present a method for scaling-up individual risk factors for heat strain to explore the population-level impacts of heat. Our model highlights high-risk groups and high-risk weather conditions in different climates. In particular, we have shown that women aged 75 and over are at greatest risk of heat strain on most days, with a clear effect of age on risk in females. Age is weaker risk determinant in males, with exposures more critical: most risk to men arises from outdoor exposure to extreme heat, with both temperature and solar radiation contributing to heat storage in this group.

Differences between males and females appear to be largely driven by sweat rates and occupations. A number of studies have shown that maximum sweat rates in women are lower than in men [47,48,49], and this component of the model leads to greater heat gain in women than men under equal conditions. Acting against this trend is the greater number of men engaged in active outdoor occupations. Using data on occupations provided by the Australian Bureau of Statistics, together with assumptions concerning behavior, we found similar numbers of males and females engaged in moderate or high exertion activities *indoors*, but four times more males than females engaged in moderate or high exertion activities *outdoors*. That is, the risk to males is largely due to their choice of occupations; women engaged in these occupations would likely face similar or greater risks of heat strain. Embedded in our model, these differences in behavior explain high risks of heat strain in working-aged men. The opposing factors of high exposure in males and lower sweat rates in females may help to explain why some studies have identified differences in risk of heat strain by gender [11] and others have not [10].

There are a number of challenges in developing this approach to modeling thermal storage. The MANMO equations at the core of the model have been derived for fit young people, and our approach extrapolates to a much wider group of individuals. Sensitivity analyses across input variables reassured us that outputs remain plausible across the population, but we do not explicitly model individual characteristics (such as percent body fat), or risk factors (such as cardiovascular disease) that may contribute to heat stress [3,52]. We have also chosen to focus primarily on work exposures when assessing risk of heat stress, although we recognize that recreational activities may also be important. We have also adopted a relatively simple model of sweating and have assumed all individuals are acclimatized; there is scope to expand this model to assume workers are not acclimatized at the start of a period of hot weather in summer, and to model interventions designed to ensure workers are acclimatized before engaging in high exertion activities. The MANMO model includes other simplifications—Such as including only a single layer of clothing—That would need modification (perhaps using adjustments as in [53]) to allow modeling of clothing-related interventions. For computational efficiency—additionally, owing to the lack of individual data—We have not modeled every individual in the population, and have made assumptions about levels of exertion for different job types. While this is not ideal, we believe it captures population-level behavior and trends. Finally, when calculating risk, we have chosen weather measurements at 3:00 p.m.; in some cases this will underestimate risk if temperature peaks much earlier.

A fundamental assumption in this paper is that individuals do not attempt to reduce their exposure to heat on hot days. Thus, our estimates reflect the maximum likely risk of heat related morbidity over an hour of heat exposure at 3:00 p.m. The next step in this project is to explore the impact of individual- and population-level interventions to reduce heat strain. By developing a mechanistic model of population risks, we are able to compare a variety of interventions, such as increasing use of air-conditioners, cooler buildings due to more heat-aware housing design, and strategies to reduce exertion during hot periods. As of 2012, around half of Australian households had reverse cycle air conditioning, with high uptake (93%) in hottest zones [54]; our model allows us to assess both national and local changes in this proportion. However, often overlooked in the limited capacity of air conditioners to cope with very hot conditions; above 32 °C, they struggle to reduce the air temperatures to desired levels [55]. Increasing prevalence of maximal temperatures exceeding 32°—indeed exceeding 40°—Therefore, renders people with air conditioners at increasing heat risk. We can also explore the likely implications of future trends—Incorporating both climate change and population changes such as rising levels of obesity. Currently, only 23% of individuals over 75 in Australia are obese [56], and it is important to assess how population levels of heat stress may change if this percentage increases. Finally, as global warming trends continue, exacerbating heat exposures and escalating health risks, we believe this work could provide an important tool for evaluating adaptation strategies to protect human health.

## 5. Conclusions

This paper presents a mechanistic model of heat strain that combines weather and individual-level risk factors to provide a population-level estimate of the risk of heat-associated illness. We show clear differences between risks in females and males, with behavior driving risks in males and reduced sweat rates a key factor in older females. Extreme heat poses a considerable risk for females aged 75 and over, while high humidity and solar radiation increases the risk of heat strain in males. In developing a population-level framework for assessing heat strain, we made certain simplifications and assumptions around both weather exposures and human behavior, and in our classifications around work-level exposures, and we acknowledge that some of these may have resulted in some misclassification of individuals. In future work, a number of potential refinements are possible, including modeling heat risks due to recreational activities, adjusting for clothing and other individual-level risk factors (such as comorbid conditions). Our eventual aim with this model is to use it to assess interventions applied at both individual and population levels. We know that many factors influence the likely uptake of health policies aimed at reducing the risk of heat stress; this model provides a tool for assessing and comparing the potential benefits of these interventions.

## Supplementary Files

Supplementary File 1
