# Supplementary material for: Estimating Risks of Heat Strain by Age and Sex: A Population-Level Simulation Model
Source: Int J Environ Res Public Health. 2015 May 18;12(5):5241–55. doi: 10.3390/ijerph120505241 (PMC4454965; doi:10.3390/ijerph120505241)
Supplement: Supplementary File 1 [file ijerph-12-05241-s001.pdf]

Supplementary Information

Estimating Risks of Heat Strain by Age and Sex: A Population-Level Simulation Model

---

Supplementary Material

This document provides additional material on the simulation model and assumptions underlying it. Figure S1 gives a representation of the model showing both individual (red) and weather-related (blue) inputs. Height and weight characteristics were sourced from the Australian Bureau of Statistics, and sweat rates were modelled from experimental data as described in the main text.

Metabolic rates and location of work (indoors/outdoors) was based on occupation data by age and sex. Occupations are typically classified according to qualification level rather than activity, and we requested data at both the major level (9 occupation classes) and the sub-major level (35 occupation classes) to be able to distinguish activities. Table S1 provides a summary table of the categories and assumptions. Each row of this table is a different occupation group, and the columns represent combinations of exposure and activity level. The 'not exposed' category refers to individuals working in air-conditioned premises where they do not experience the outdoor temperature. Other categories are combinations of indoor or outdoor exposure together with four exertion levels: Rest, minimal exertion, moderate exertion and heavy exertion. Metabolic rates are calculated from these levels of exertion assuming that a 70 kg individual has metabolic rate of 125 W at rest, 225 W during minimal exertion, 600 W during moderate exertion, and 1280 W during heavy exertion.

Figures S2 and S3 show the impact of individual characteristics and weather variables on heat storage and hourly temperature gain in an individual. In each graph, other parameters are kept (largely) fixed, and one parameter is varied across a plausible range. Figure S2 shows a slight increase in hourly temperature gain with increasing mass (where height was fixed), and a slight decrease in hourly temperature gain with increasing height (where mass was kept fixed). Hourly temperature gains increase with exertion levels, and decreased with maximal sweat rates, as expected. While both temperature and relative humidity increase heat storage, graphs show a threshold at which the rate of heat storage increases more rapidly (Figure S3). These thresholds differ between temperature and humidity and for individuals indoors and outdoors.

Table S1. Exposure and exertion assumptions by occupation class, with color indicating increasing metabolic rate.

	Not Exposed [1]	Rest (No Exertion)		Minimal Exertion		Moderate Exertion		Heavy Exertion	
		in	out	in	out	in	out	in	out
Managers	100%								
Professionals	100%								
Technicians and Trade Workers Indoors includes: ICT, Science, and Food trades workers.	40%			30%		30%			
Technicians and Trade Workers Outdoors includes: Automotive, Animal, Construction and Horticultural Workers.	20%				45%		30%		5%
Community and Personal Service Workers Indoors includes: Health and Welfare, Carers and Aides, and Hospitality workers.	40%			30%		30%			
Community and Personal Service Workers Outdoors includes: Sports and Personal Service, and Protective Service workers.	20%				40%		40%		
Clerical and Administrative Workers	50%	50%							
Sales Workers	100%								
Machinery Operators and Drivers includes: Machine operators, Road and Rail drivers.		20%	20%	20%	20%		20%		
Storepersons	80%			20%					
Cleaners and Laundry workers				20%		40%		40%	
Construction, Mining, Farm, and Garden Labourers				30%	10%	10%	25%		25%
Factory, and Food Preparation Labourers				50%		50%			
Not employed	40%	15%	5%	15%	5%	10%	5%	3%	2%

[1] Individuals not exposed are assumed to work in an air-conditioned environment.

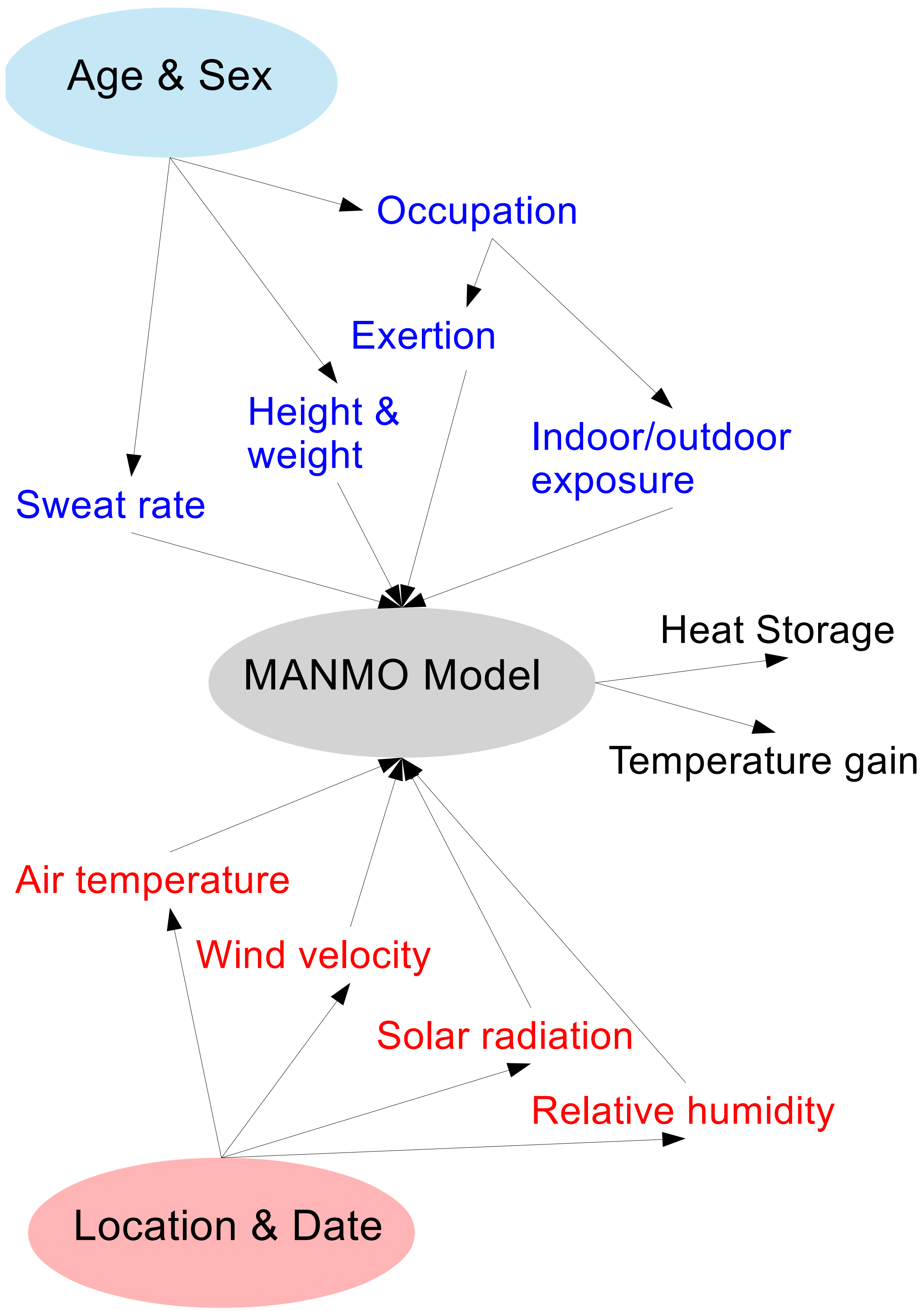

Figure S1. Model diagram showing the influence of location and date on weather variables (**Red**), and of age and sex on individual risk characteristics (**Blue**) all included in the MANMO model, which provides output of either temperature gain or heat storage.

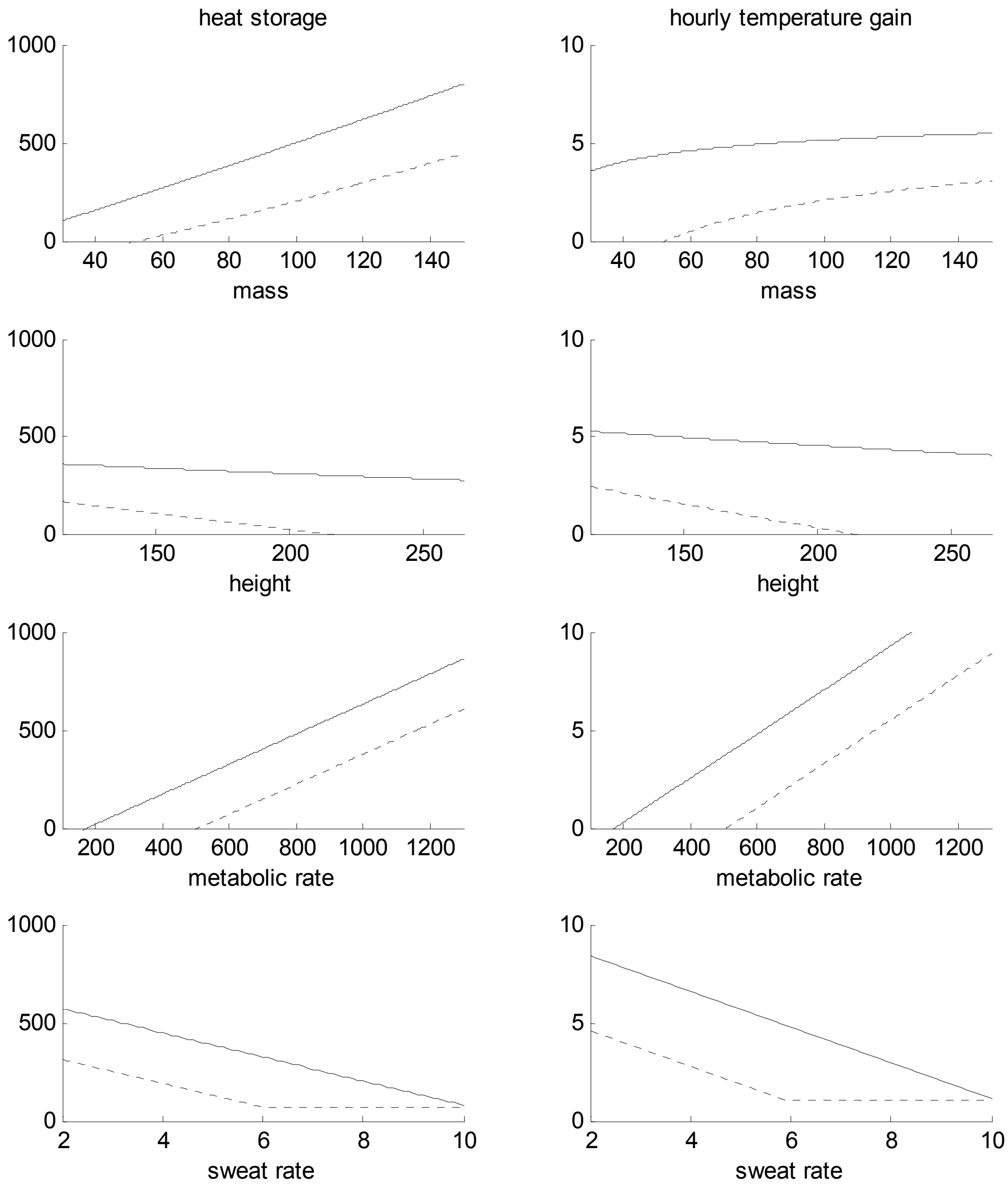

Figure S2. Impact of individual characteristics on heat storage and hourly temperature gain in individuals outdoors (solid line) and indoors (dotted line). Other parameters largely fixed as: mass of 70 kg height of 170 cm, metabolic rate of 600 W, sweat-rate of 6 $g \cdot m^{-2} \cdot min^{-1}$, temperature of 38 °C, relative humidity of 40%, and solar radiation of 305 $W \cdot m^{-2}$, except for an adjustment to the metabolic rate with mass as in Weyand 2009 (see main text for more detail).

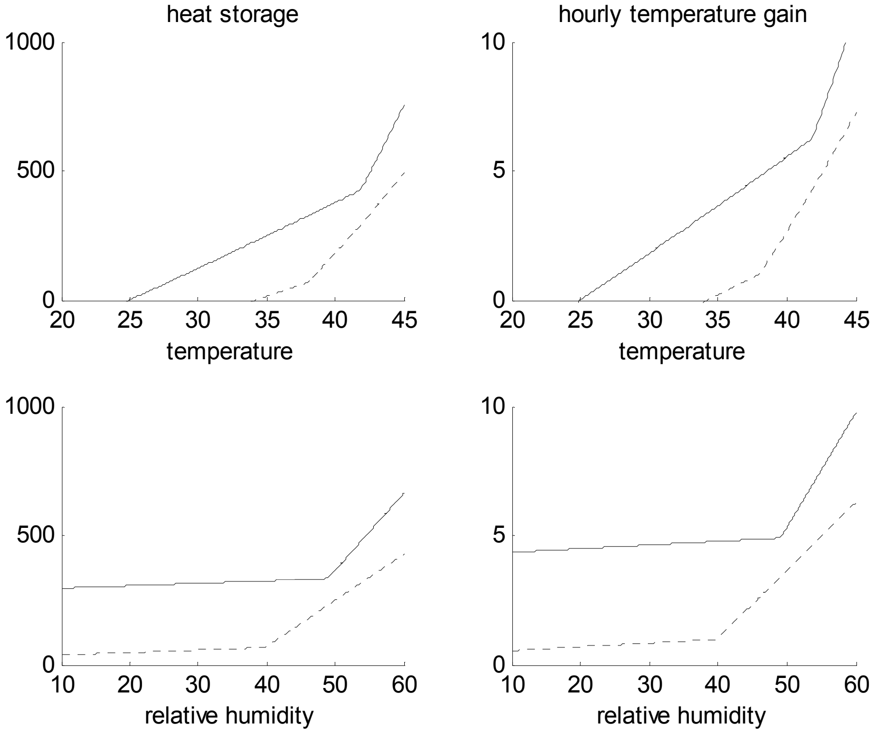

Figure S3. Impact of weather conditions on heat storage and hourly temperature gain in individuals outdoors (solid line) and indoors (dotted line). All other parameters are kept fixed as: mass of 70 kg, height of 170 cm, metabolic rate of 600 W, sweat-rate of 6 $g{\cdot}m^{-2}{\cdot}min^{-1}$, temperature of 38 °C, relative humidity of 40%, and solar radiation of 305 $W{\cdot}m^{-2}$.

© 2015 by the authors; licensee MDPI, Basel, Switzerland. This article is an open access article distributed under the terms and conditions of the Creative Commons Attribution license (http://creativecommons.org/licenses/by/4.0/).
